# Relationship Between Overnight Dexamethasone Suppression Test and Aging

**DOI:** 10.7759/cureus.48383

**Published:** 2023-11-06

**Authors:** Serpil Ciftel, Filiz Mercantepe

**Affiliations:** 1 Department of Endocrinology and Metabolism, Faculty of Medicine, Erzurum Regional Training and Research Hospital, Erzurum, TUR; 2 Department of Endocrinology, Diabetes and Metabolism, Faculty of Medicine, Recep Tayyip Erdogan University, Rize, TUR

**Keywords:** pituitary incidentaloma, adrenal gland incidentaloma, elderly population, dexamethasone suppression test, aging, adrenal glands

## Abstract

Background

This study aims to investigate the relationship between suppressed cortisol levels measured after the 1-mg dexamethasone suppression test (DST) and age based on the hypothesis that aging can alter the activity of the hypothalamic-pituitary-adrenal (HPA) axis.

Methodology

Data obtained by the retrospective evaluation of suppressed 1-mg overnight DST results of adults aged ≥18 years with adrenal incidentaloma or suspected endogenous hypercortisolemia between December 2021 and March 2023 were subjected to age-dependent correlation analysis. Individuals aged between 18 and 90 years (n = 1111) were classified into the following four groups: <30 years, 30-49 years, 50-69 years, and >70 years. DST results were compared according to age groups.

Results

Median post-DST cortisol was 18.49 nmol/L, with a level of 17.9 nmol/L in females and 20.7 nmol/L in males. The overall rate of DST suppression was 62.7%, with a rate of 63.8% in females and 59.7% in males. On pairwise comparisons of all age groups, there was a difference in post-DST cortisol levels (p = 0.000). Our statistical analysis revealed a strong positive correlation between age and cortisol levels after DST.

Conclusions

The negative feedback mechanism for cortisol may be altered in older patients. Therefore, the 1-mg DST may yield a higher rate of false positives in the elderly.

## Introduction

The 1-mg overnight dexamethasone suppression test (DST) is the primary test when hypercortisolemia is suspected in adults [1-3]. According to the European Society of Endocrinology (ESE), a 1-mg DST should be given to all people who have an adrenal incidentaloma or who show signs of endogenous hypercortisolemia [3]. DST is a reliable and inexpensive screening test with an easy application [4,5]. The test is applied by administering the patient 1 mg of dexamethasone at night and instructing them to fast overnight, after which serum cortisol levels are measured between 08:00 and 09:00 in the morning [5,6]. In those with post-DST serum cortisol levels ≤1.8 μg/dL (50 nmol/L), the test is considered suppressed with 95% sensitivity and 80% specificity [7,8]. Low-dose DST usually does enough to block the hypothalamic-pituitary-adrenal (HPA) axis. However, it can provide wrong or misleading results if someone is overweight, depressed, psychotic, pregnant, or taking drugs that change cortisol-binding globulin levels or the metabolism of dexamethasone [9].

The elderly population is rapidly rising worldwide [10]. An increase in the prevalence of adrenal incidentaloma is concurrent with this rise [10]. To check for adrenal incidentaloma, all adults are administered a standard dose of 1 mg dexamethasone for the 1-mg overnight DST [3]. The test does not have different dexamethasone doses for different age ranges or age-dependent cutoff values. However, drug responses are known to vary in older individuals due to pharmacokinetic and pharmacodynamic changes [11]. As people get older, their organ function, receptor responses, body composition, nutrition, comorbid diseases, and medication use change from what they were like when they were young [11]. DST metabolism and absorption also have an impact on the results [8]. In addition, the hypothalamic-pituitary-adrenal (HPA) axis also changes with aging [12]. In older individuals, the sensitivity of the HPA axis decreases, and the negative feedback response to cortisol declines [13]. Moreover, the timing of the morning peak of cortisol shifts to an earlier time in the elderly [14]. This difference stimulated an interest in understanding the rates of change in DST, which is used for the diagnosis of hypercortisolemia, in older individuals. For this purpose, we retrospectively analyzed cortisol levels after suppressing DST according to gender and age groups.

## Materials and methods

We conducted this retrospective study by examining the 1-mg overnight DST results of adults aged ≥18 years who had adrenal incidentaloma or suspected endogenous hypercortisolemia in the outpatient clinic of Recep Tayyip Erdoğan University, Faculty of Medicine, Division of Endocrinology, between December 2021 and March 2023. The relevant clinical data for this study were obtained through an examination of electronic medical records. The Institutional Review Board of Recep Tayyip Erdogan University approved the study protocol (approval number: E-40465587-050.01.04-725). This study was conducted in accordance with the guidelines of the Helsinki Declaration.

The center where the study was conducted was a tertiary hospital and followed a standard DST protocol conforming to the ESE guidelines. The test methodology (administration of 1 mg dexamethasone at 23:00 overnight, providing fasting blood samples at 08:00-09:00 in the morning) was described to the patient by their doctor in detail. Exclusion criteria are presented in Table 1. Serum cortisol levels were measured with the enzyme-linked immunosorbent assay method. Cortisol measurements were presented as nanomoles per liter (nmol/L). DST was considered to be suppressed in those with serum cortisol levels ≤50 nmol/L (1.8 μg/dL). Individuals whose serum cortisol levels were above 50 nmol/L were given second-line tests to ensure they did not have hypercortisolism. In addition, pheochromocytoma and primary hyperaldosteronism were excluded in all patients with adrenal incidentaloma.

**Table 1:** Criteria for excluding participants from the study.

Exclusion criteria
Primary hyperaldosteronism
Pheochromocytoma
Pregnant women
Lactation
Unregulated diabetes mellitus
Unregulated hypertension
Using oral contraceptives
Using drugs affecting the dexamethasone metabolism
Psychiatric disorders such as schizophrenia

A total of 1,267 individuals underwent the 1-mg overnight DST for adrenal incidentaloma or suspicion of endogenous hypercortisolemia between December 2021 and March 2023. A cutoff point of 50 nmol/L for the cortisol level after DST was the threshold for cortisol excess. DST >50 nmol/L was reported in 156 individuals (12.3%). These 156 individuals were excluded to eliminate potential confounding factors. The results of the remaining 1,111 individuals (865 females and 246 males) with DST ≤50 nmol/L were evaluated based on gender and age ranges. Patients were classified into the following four age groups to be evaluated based on age: <30 years (n = 294), 30-49 years (n = 443), 50-69 years (n = 335), and >70 years (n = 39). In our study, cortisol suppression levels were calculated as % using a threshold value of 50, as the patients did not have a basal cortisol level. The percentage of suppression was calculated using the following formula: (50-cortisol level) × 100/50.

Statistical analysis

SPSS version 22.0 (IBM Corp., Armonk, NY, USA) statistical software was used for all analyses. Visual and analytical methods (Kolmogorov-Smirnov/Shapiro-Wilk test) were used to examine the variables and determine if they were normally distributed. We used the Kruskal-Wallis test to compare non-normally distributed variables. Pairwise comparisons were made with Dunn’s test. We used Spearman’s correlation coefficient for correlation analysis. P-values <0.05 were deemed statistically significant.

## Results

This study included 1,111 patients with a median age of 42 (18-90) years, consisting of 865 females (78%) with a median age of 41 (18-90) and 246 males (22%) with a median age of 45 (18-90) (Table 2). Females had a significantly lower mean age than males. Different people had different reasons for undergoing DST, such as to check for an adrenal mass, a hypophyseal mass, or Cushing syndrome. Overall, the median cortisol level after DST was 18.49 (2.76-49.4) nmol/L. In women, it was 17.9 (2.76-44.08) nmol/L, and in men, it was 20.7 (11.04-49.4) nmol/L (Tables 2, 3). Post-DST cortisol levels were higher in males compared to females, with statistical significance (p = 0.033) (Table 3). The overall rate of DST suppression was 62.7% (0.56-94.4), with 63.8% (0.56-94.4) in females and 59.7% (0.56-77.7) in males when a cortisol level of 50 nmol/L was considered the basal cortisol value. Females had a higher DST suppression rate than males (p = 0.33) (Table 3).

**Table 2:** Characteristics of the patients in the study group in terms of investigated parameters. Non-normally distributed variables are presented as median (minimum-maximum).

	Age (year)	Cortisol level (nmol/L)	Cortisol suppression rate (%)
Study group (n = 1,111)	42 (18–90)	18.49 (2.76–49.4)	62.7 (0.56–94.4)
Sex n (%)			
Female (n = 865)	41 (18–90)	17.9 (2.76–46.08)	63.8 (0.56–94.4)
Male (n = 246)	45 (18–90)	20.7 (11.04–49.4)	59.7 (0.56–77.7)

**Table 3:** Comparisons of post-DST cortisol levels with gender (median (minimum-maximum)). *: p ≤ 0.05 is significant. Mann-Whitney U test. DST: dexamethasone suppression test

	Female (n = 865)	Male (n = 246)	P-value
Cortisol levels after DST (nmol/L)	17.9 (2.76–49.4)	19.86 (11.04–49.4)	0.033*
Cortisol suppression levels (%)	63.8 (0.56–94.4)	59.7 (0.56–77.7)	0.033^*^

The 1,111 participants were divided into four groups as follows: <30 years (n = 294), 30-49 years (n = 443), 50-69 years (n = 335) and >70 years (n = 39). Age groups were compared in terms of cortisol and suppression percentages after DST. Individuals aged <30 years had a DST value of 16 nmol/L (2.76-48.8) and a DST suppression rate of 67.7% (1.67-94.4), individuals aged 30-49 years had a DST of 16.83 nmol/L (2.76-44.14) and a DST suppression rate of 66.1% (11.1-94.4), individuals aged 50-69 years had a DST of 24 nmol/L (11.04-49.4) and a DST suppression rate of 51.6% (0.56-77.7), and individuals aged >70 years had a DST value of 35.04 nmol/L (16-49.4) and a DST suppression rate of 29.4% (0.56-67.7) (Table 3). The lowest cortisol level after DST was found in the age group below 30, while the highest level was found in the group over 70. In the pairwise comparisons of all age groups, there was a difference between all groups in terms of post-DST cortisol levels (p < 0.001) (Table 4).

**Table 4:** Comparison of post-DST cortisol levels and suppression percentages between age groups (Median-(min-max)). Paired comparisons were made between groups with independent-sample Kruskal-Wallis test. DST: dexamethasone suppression test

	<30 (n = 294)	Years 30–49 (n = 443)	50–69 (n = 335)	>70 (n = 39)	Total (n = 1,111)	P-value
Age (year)	23 (18–29)	40 (30–49)	57 (50–69)	75 (70–90)	42 (18–90)	0.001
Cortisol after DST	16 (2.76–48.8)	16.83 (2.76–44.14)	24 (11.04–49.4)	35.04 (16–49.4)	18.49 (2.76–49.4)	0.001
Suppression (%)	67.7 (1.67–94.4)	66.1 (11.1–94.4)	51.6 (0.56–77.7)	29.4 (0.56–67.7)	62.7 (0.56–94.4)	0.001
Pairwise comparison with the other three groups*	0.001	0.001	0.001	0.001	0.001	0.001

**Figure 1:**
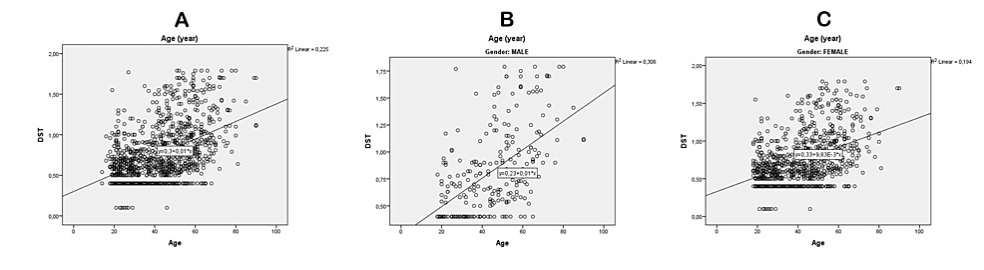
(A) Correlation of post-DST cortisol levels with age. (B) Correlation of post-DST cortisol levels with age in the male gender. (C) Correlation of post-DST cortisol levels with age in the female gender. DST: dexamethasone suppression test

Our statistical analysis revealed a strong positive correlation between age and cortisol levels after DST (r = 0.447, R^2^ = 0.225, p < 0.001) (Table 5, Figure 1A). However, this correlation was found to be stronger in males (r = 0.568, R^2^ = 0.306, p < 0.001) (Table 5, Figure 1B) than in females (r = 0.405, R^2^ = 0.194, p < 0.001) (Table 5, Figure 1C). Additionally, the percentage of cortisol suppression was found to be higher in younger patients and lower in older patients (Table 4).

**Table 5:** Spearman correlation analysis between age and cortisol level after DST. Correlation is significant at the *p < 0.01 level. DST: dexamethasone suppression test

Age	r	R^2^	P-value*
Cortisol (total)	0.447	0.225	0.001
Female	0.405	0.194	0.001
Male	0.568	0.306	0.001
Supression (%)	-0.447		0.001
Female	-0.405		0.001
Male	-0.568		0.001

## Discussion

This study revealed higher suppressed cortisol levels after the 1-mg DST in older individuals. The results of this study show that older individuals have lower DST suppression rates compared to young adults. Except for a few studies suggesting that age and DST are not related, data from the literature have reported higher post-DST cortisol values in older individuals compared to young adults [8,15,16]. Our results are consistent with the literature. Two previous studies evaluating data from 278 and 402 obese patients regarding low-dose DST demonstrated a positive correlation between cortisol levels after the 1-mg DST and age [16,17]. However, their studies included non-suppressed DST data (post-DST cortisol >50 nmol/L). In a study by Castro et al. that evaluated 593 adults with non-functioning adrenal incidentaloma, those with DST >1.4 µg/dL were older than those with ≤1.4 µg/dL [18]. They stated that this was probably due to the age-dependent changes in the sensitivity of the HPA to both exogenous and endogenous stimulants.

Although the changed cortisol axis in older individuals has been examined in previous studies, the mechanism of the change that occurs in glucocorticoid metabolism has not been clarified. Due to the limited number of studies, information regarding the changed HPA axis in old age has remained hypothetical. DST relies on the negative feedback mechanism of the HPA axis [17]. With aging, the HPA axis undergoes changes [19]. Aging causes changes in both adrenocorticotropic hormone (ACTH) and cortisol secretion and the adrenal gland [14]. As we age, the HPA axis becomes less sensitive, which means that the negative feedback in cortisol secretion is less strong [20]. In the elderly, daily serum cortisol levels and cortisol production rate are generally determined to be higher [19]. Twenty-four-hour total and free plasma and saliva cortisol were elevated in the elderly [13]. The morning peak of cortisol shifts to earlier hours compared to younger individuals [21]. Cortisol levels are especially higher in the evening and at night with aging relative to younger individuals [13]. Therefore, higher dexamethasone doses may be needed to achieve suppression of cortisol in the elderly.

The HPA axis may become active with age as a natural part of getting older, or it may become active because of hypercortisolism, a condition linked to diseases that come with getting older. The higher cortisol levels that come with getting older may cause many changes and bad effects in an older person’s body. On the other hand, the older person’s systems may change, they may have comorbid diseases, and they may be more fragile. The neurodegenerative changes occurring along with physiological aging may be responsible for the disruption of the sensitivity to glucocorticoid feedback and the increased activation of the HPA axis [13,14]. Studies have found a negative link between age and cortisol in terms of the size of the hippocampi and amygdala and the thickness of the caudal anterior cingulate cortex [22]. In healthy older adults, higher levels of cortisol after DST were mostly linked to a smaller left anterior cingulate cortex volume [22]. Senile dementia was found to be associated with certain changes in the HPA axis, and an increase in cortisol activity was determined [10]. As we do not have data on the cognitive function of the older population included in our study, we are not able to interpret our results in this respect.

Older individuals are different from young adults in multiple aspects. Primarily, aging reduces adaptation to environmental changes and conditions of stress [15]. The level of frailty increases in the elderly. The increase in stress may be responsible for elevated cortisol levels. Moreover, certain changes in body composition appear with aging. The elderly experience a decrease in muscle mass and an increase in visceral fat mass [11]. The conversion of corticosterone into cortisol in adipose tissue increases [10]. Studies have linked increased fat mass and decreased muscle mass in the elderly to elevated cortisol levels [20]. In addition, total body fluid declines with aging, and its distribution volumes are altered. At the same time, kidney function shows a 1 mL/minute/1.73 m^2^ decrease in glomerular filtration rate each year after the age of 40 [11]. Incomplete suppression of plasma cortisol after DST has been reported in chronic kidney failure [23]. Moreover, with aging, type 1 11β-hydroxysteroid dehydrogenase activity increases in certain tissues, contributing to the increase in cortisol production [10]. A decrease in the liver mass of older individuals is also encountered [24]. The form of cortisol is free at a rate of 6%, cortisol-binding globulin (CBG)-bound at a rate of 80%, and albumin-bound at a rate of 14% [18]. The majority of CBG is synthesized in the liver, and an aging-related change in CBG levels has not been reported [21]. A decrease in liver mass or certain drugs can modify the CYP3A4 enzyme metabolism [1,7]. The individuals included in our study were not on medications that affected the CYP3A4 enzyme metabolism.

Dexamethasone is used to assess the negative feedback of the HPA axis as it is a strong synthetic glucocorticoid that is not affected by 17-hydroxysteroids [25]. DST is a screening test that has acceptable false-negative rates and considerable false-positive rates [26]. Several conditions, including depression, psychosis, the use of medications influencing the dexamethasone metabolism, and, most primarily, obesity, are known to result in false-positive outcomes [27]. In addition, some studies have reported inadequate dexamethasone concentrations to result in false positivity at rates between 6% and 20% [8,28-30]. The pharmacokinetic and pharmacodynamic properties of medications are different in the elderly [11]. The peak concentration of medications and the timing of the peak are altered [11]. Organ dysfunctions, gastrointestinal diseases, reduced gastric acid secretion, reduced absorption surface, and slowed gastric emptying may lead to inadequate dexamethasone concentrations [1,4,5]. Dexamethasone clearance increases in the presence of chronic kidney diseases [23]. All of these conditions may result in inadequate dexamethasone concentrations, and, hence, false-positive or less suppressed cortisol results on DST. Probably with the contribution of all of the factors mentioned above, dexamethasone fails to inhibit cortisol as effectively in the elderly as it does in young individuals. In older individuals, higher dexamethasone doses may be needed to suppress endogenous cortisol and reduce false positivity rates.

Along with aging, the body mass index also determines the adrenal sensitivity to ACTH [16]. As we could not determine the body mass indices of the patients due to the retrospective nature of our study, we were not able to include them in our statistical analysis. Therefore, we cannot comment on the contribution of BMI to the age-dependent suppression levels of DST. Moreover, DST indications were variable in our study, and the distribution rates of these indications could not be determined. Therefore, we were not able to form an opinion as to whether there are changes in DST suppression rates according to the indications. We did not include patients with post-DST cortisol >50 nmol/L (1.8 μg/dL) to eliminate confounding factors that could lead to false positivity, such as patient non-compliance, and to exclude patients with true hypercortisolemia.

The results of our study showed higher post-DST cortisol levels in males. Data from the literature on gender-related cortisol and DST results are conflicting [14,20-30]. In a study by Purnell et al., cortisol production rate and free cortisol levels were found to be significantly higher in males than in females [20]. Studies have also reported that post-DST cortisol and dexamethasone levels do not change between genders [8]. However, the effectiveness of ACTH decreases in males and increases in females with age [14,21]. The results of our study revealed a stronger positive correlation between post-DST cortisol levels and age in males than in females. This is probably because our male cohort had a more advanced age.

The results of this study are important in the following regards: it may predict more frequent false-positive DST results in older individuals. The use of higher dexamethasone doses in the application of DST may potentially reduce false-positive test results in older individuals. On the other hand, this study should be interpreted with consideration of certain strengths and limitations. The standard application of DST in our clinic according to a specific procedure and the relatively sufficient sample size constitute the strengths of this study. As our study had a retrospective design, we were not able to access certain data that could serve as confounding factors, such as DST indications, body mass indices, medication use, psychiatric disorders, and comorbid diseases, due to the nature of the study. Additionally, as we only evaluated the negative feedback mechanism of the HPA axis, it would be speculative to state that the cortisol metabolism undergoes a complete change in older individuals. There were fewer male patients and patients >70 years old. The results were collected from a single center.

## Conclusions

Response to the 1-mg overnight DST declines with aging. More research is needed to find a higher cutoff value for measuring cortisol or to give older people a higher dose of dexamethasone to lower the number of false-positive DST results. Based on the results of this study, we recommend an age-range-dependent approach to DST in clinical practice. The potentially higher false-positive rate should be kept in mind in cases where DST is not suppressed in elderly patients with suspected cortisol excess. We suggest carefully evaluating geriatric patients to avoid second-line testing and unnecessary procedures.
